# Alpha-fetoprotein predicts the treatment efficacy of immune checkpoint inhibitors for gastric cancer patients

**DOI:** 10.1186/s12885-024-11999-z

**Published:** 2024-02-26

**Authors:** Jingjing Zhang, Lei Wang, Shasha Zhang, Ruijie Cao, Yufei Zhao, Yue Zhao, Yanrong Song, Zhanjun Guo

**Affiliations:** 1 Department of Immunology and Rheumatology, The Fourth Hospital of Hebei Medical University, 12 Jiankang Road, 050011 Shijiazhuang, Hebei P.R. China; 2 Department of Thoracic Surgery, The Fourth Hospital of Hebei Medical University, 050011 Shijiazhuang, Hebei P.R. China; 3 Department of Medical Technology, Xingtai Medical College, 054000 Xingtai, Hebei P.R. China

**Keywords:** Gastric cancer, Alpha-fetoprotein, Immune checkpoint inhibitors, Anti-PD-1 antibody, Therapeutic efficacy

## Abstract

**Background:**

Immune checkpoint inhibitors (ICIs) are commonly used in conjunction with chemotherapy to improve treatment outcomes for patients with gastric cancer. Since AFP could influence immunity by both inhibiting natural killer (NK) cells and regulating negatively the function of dendritic cells, we evaluated the influence of baseline serum alpha-fetoprotein (AFP) levels on the curative effect of ICIs in advanced gastric cancer (AGC) patients.

**Methods:**

A retrospective analysis was conducted on 158 AGC patients who underwent ICI treatment. The patients were divided into high and low groups based on the AFP threshold of 20 ng/ml. The efficacy of ICI treatment was assessed using objective response rate (ORR), disease control rate (DCR), progression-free survival (PFS), and overall survival (OS).

**Results:**

The higher levels of baseline AFP were found to be associated with a decrease in the effectiveness of ICIs, as evidenced by a DCR of 50.0% in the group with high AFP levels compared to 87.7% in the group with low AFP levels (*P* < 0.001). Further analysis using Kaplan-Meier survival techniques indicated that a high AFP level was linked to shorter progression-free survival (PFS) (*P* < 0.001) and overall survival (OS) (*P* = 0.001) in AGC individuals receiving ICIs. After propensity score matching, a log rank test revealed that the high AFP group had a decrease in median PFS (*P* = 0.011) and median OS (*P* = 0.036) compared to the low AFP group. The high AFP levels also showed its association with shorter PFS and OS in the subgroup analysis of ICI plus chemotherapy patients.

**Conclusions:**

Baseline AFP levels may predict immune checkpoint inhibitor treatment efficacy in AGC patients.

**Supplementary Information:**

The online version contains supplementary material available at 10.1186/s12885-024-11999-z.

## Introduction

Gastric carcinoma (GC) remains a common type of lethal malignancy worldwide. According to GLOBOCAN’s latest released data, there were more than 1,000,000 novel cases of GC and an approximate 769,000 deaths in the year 2020. This places GC as the fifth most frequently occurring cancer and the fourth leading cause of mortality among all malignant tumors [1]. The risk factors associated with the initiation of gastric cancer include the presence of Helicobacter pylori (Hp) infection, the consumption of preserved foods, the intake of alcoholic beverages, and tobacco usage [1]. Surgical intervention remains the primary approach for achieving curative treatment of GC. In addition, there are alternative treatment options available, such as chemotherapy, radiotherapy, molecular targeted therapy, and immunotherapy. Of these, systemic chemotherapy serves as the main therapeutic modality for advanced gastric cancer (AGC) [2]. Additionally, targeted therapies like trastuzumab (for HER-2 positive patients), apatinib, and ramucirumab have demonstrated efficacy in improving the life expectancy of AGC patients [3]. Despite these advancements, the prognosis for AGC patients remain poor with median survival times ranging from 10 to 13 months [4]. Immune checkpoint inhibitors (ICIs) trigger antitumor activity by blocking intrinsic downregulating factors of the immune system such as programmed cell death 1 (PD-1), programmed cell death ligand 1 (PD-L1), and cytotoxic T-lymphocyte antigen 4 (CTLA-4), have shown significant breakthroughs in treating different types of cancer, including melanoma, gastric carcinoma, and non-small cell lung cancer [5–7]. The blocking of the PD-1/PD-L1 signaling pathway by PD-1/PD-L1 inhibitors can effectively enhance the function of T-lymphocytes, resulting in the promoting of anti-tumor immunity, suppressing of tumor immunity, and suppressing of tumor growth [6, 8]. In clinical trials ATTRACTION-4 and CheckMate 649, the combination of nivolumab, an anti-PD-1 monoclonal antibody, with chemotherapy has showed significantly longer progression-free survival (PFS), a higher disease control rate (DCR), and a higher objective response rate (ORR), thus leading to its approval as a first-line treatment for advanced gastric cancer [6, 9]. Additionally, other anti-PD-1 agents have also shown clinical benefits when combined with molecular targeted therapy or chemotherapy in treating advanced gastric cancer [10, 11]. However, despite these notable breakthroughs, only a limited number of predictive biomarkers for the efficacy of immunotherapy in AGC, such as PD-L1 expression, microsatellite instability (MSI)/mismatch repair (MMR), and gut microbiota, have been uncovered in previous studies [12–14].

Αlpha-fetoprotein (AFP) is a monosaccharide protein primarily synthesized from the fetal liver and the yolk sac during fetal development [15]. Elevated serum AFP levels have been observed in solid tumors of various other organs, including the stomach, pancreas, colon, gallbladder, and lung [16–19]. AFP elevation in GC is the most common condition in the extrahepatic tumors [16]. AFP has various biological functions that not only acts as a tumor marker but also regulates cell proliferation, differentiation, and tumor formation [20]. Moreover, there have been reports that AFP has an immune suppressive function by inhibiting natural killer (NK) cells or negatively regulating the function of dendritic cells [21]. Several retrospective studies have indicated that the baseline serum AFP level and early treatment response of AFP were related to treatment efficacy and prognosis of ICIs for hepatocellular carcinoma patients [22, 23]. However, whether the serum AFP level affects the prognosis of ICIs treatment for AGC patients is still unknown. Therefore, we performed a retrospective analysis to evaluate the prognostic role of the baseline serum AFP level in AGC patients receiving ICIs treatment.

## Methods

### Patients

The participants involved in this study were patients diagnosed with advanced gastric cancer (AGC) who were treated with anti-PD-1 antibody at The Fourth Hospital of Hebei Medical University between January 2019 to September 2023. Patients who had previously undergone immunotherapeutic treatments were excluded from the study. Various clinical information was collected retrospectively for analysis, including the patients’ gender, age, Eastern Cooperative Oncology Group Performance Status (ECOG PS), combined positive score (CPS), status of human epidermal growth factor receptor 2 (HER2), status of Epstein-Barr virus (EBV), TNM staging, surgical history, treatment regimen, treatment lines, MSI status, liver metastases, disease status, and baseline AFP levels. Serum AFP levels were analyzed using an AFP detection kit (Roche Diagnostics, Basel, Switzerland), and a threshold of 20 ng/ml was used to define the low (≤ 20 ng/ml) and high (> 20 ng/ml) groups [24, 25]. The baseline AFP level was determined as the AFP value prior to the initiation of immunotherapy. All experimental procedures were reviewed and approved by the Fourth Hospital of Hebei Medical University Ethics Committee(No. 2,021,136). Since this study was conducted retrospectively using only existing information, the requirement for informed consent was waived by the Fourth Hospital of Hebei Medical University Ethics Committee.

### Treatment and evaluation

The patients were treated with anti-PD-1 antibodies (either alone or in combination with chemotherapy/targeted therapy) every three weeks until there was evidence of disease progression, clinical decline, intolerable toxicity, or withdrawal of consent. Tumor assessments were performed using magnetic resonance imaging or computed tomography scans every two to three cycles, following the RECIST criteria version 1.1 until tumor progression [26]. The study included the evaluation of progression-free survival (PFS), overall survival (OS), and tumor response.

### Statistical analysis

The statistical analysis was conducted by SPSS Statistics 21.0 (IBM SPSS, NY, USA). PFS was the duration between the initiation of anti-PD-1 therapy and the occurrence of progressive disease, death, or the study’s cutoff point. OS was the duration between the start of ICI treatment and death or the study’s cutoff point. The analysis of enumeration data was performed using either the χ2 test or Fisher Exact test. Survival curves were generated using the Kaplan-Meier method, and the relationship between clinical characteristics and survival was assessed using the log-rank test. The multivariable survival evaluation was performed using the Cox proportional hazard model. To balance the differences in baseline characteristics between the two groups, we calculated propensity scores for clinical characteristics with stata15 (64-bit) to decrease the effect of potential confounding factors. A *P*-value below 0.05 was deemed to be statistically noteworthy.

## Results

### Patient characteristics

A total of 158 participants diagnosed with AGC and treated with anti-PD-1 antibodies (anti-PD-1 Abs) were included in this research. Among them, 30 individuals underwent a combination of immunotherapy, targeted therapy, and chemotherapy, 18 received immunotherapy plus targeted therapy, and 110 received immunotherapy alongside chemotherapy. The clinical features of the individuals have been provided in Table 1. The overall median PFS and OS were found to be 12.000 months (95% CI: 9.769–14.231 months) and 19.267 months (95% CI: 15.973–22.560 months), respectively. In terms of efficacy, one patient experienced a complete response (CR), twenty-five patients experienced a partial response (PR) and one hundred and five patients experienced stable disease (SD) (Table 2), which resulting in an Objective response rate (ORR) of 16.5% (95% CI: 10.6-22.3%) and a Disease control rate (DCR) of 82.9% (95% CI: 77.0-88.8%).

**Table 1:** Characteristics of advanced gastric cancer patients with different AFP levels

Covariate	Total No. (%)	Low AFP (%)	High AFP (%)	***P***
Total	158	138	20	
Age				
< 60	54(34.2)	48(34.8)	6(30.0)	0.673
≥ 60	104(65.8)	90(65.2)	14(70.0)	
Gender				
Male	120(75.9)	102(73.9)	18(90.0)	0.196
Female	38(24.1)	36(26.1)	2(10.0)	
ECOG PS				
0–1	92(58.2)	83(60.1)	9(45.0)	0.199
2–3	66(41.8)	55(39.9)	11(55.0)	
CPS				
< 5	117(74.1)	99(71.7)	18(90.0)	0.181
≥ 5	41(25.9)	39(28.3)	2(10.0)	
HER2 status				
Negative	133(84.2)	116(84.1)	17(85.0)	1.000
Positive	25(15.8)	22(15.9)	3(15.0)	
EBV status				
Negative	144(91.1)	124(89.9)	20(100.0)	0.284
Positive	14(8.9)	14(10.1)	0(0.0)	
Surgical history				
No	110(69.6)	95(68.8)	15(75.0)	0.576
Yes	48(30.4)	43(31.2)	5(25.0)	
TNM stage				
III	55(34.8)	51(37.0)	4(20.0)	0.137
IV	103(65.2)	87(63.0)	16(80.0)	
Treatment regimen				
ICI plus chemotherapy	110(69.6)	97(70.3)	13(65.0)	0.092
ICI plus targeted therapy	18(11.4)	13(9.4)	5(25.0)	
ICI plus chemotherapy and targeted therapy	30(19.0)	28(20.3)	2(10.0)	
Treatment lines				
1–2	138 (87.3)	123(89.1)	15(75.0)	0.157
≥ 3	20(12.7)	15(10.9)	5(25.0)	
Liver metastases				
No	116(73.4)	106(76.8)	10(50.0)	0.011
Yes	42(26.6)	32(23.2)	10(50.0)	

AFP: alpha-fetoprotein; ECOG PS: Eastern Cooperative Oncology Group Performance Status; CPS: Combined Positive Score; HER2: human epidermal growth factor receptor 2; EBV: Epstein–Barr virus

**Table 2:** Response to immunotherapy

Response	Total No.	Low AFP group	High AFP group	***P***
PD	27	17	10	
SD	105	99	6	
PR	25	21	4	
CR	1	1	0	
ORR	16.5% (95% CI: 10.6-22.3%)	15.9% (95% CI: 10.5-23.6%)	20% (95% CI: 0.8-39.2%)	0.995
DCR	82.9% (95% CI: 77.0-88.8%)	87.7% (95% CI: 82.1-93.2%)	50% (95%CI: 26.0-74.0%)	0.000

CR: complete response; PR: partial response; SD: stable disease; PD: progressive disease; DCR: disease control rate; ORR: objective response rate; AFP: alpha-fetoprotein

### Association between AFP and DCR in AGC patients

As described in Table 1, the clinical features including age, gender, ECOG PS, CPS, HER2 status, EBV status, TNM stage, surgical history, treatment regimen, treatment lines, and disease status of the two groups were comparable. Ten patients (50%) displayed the liver metastasis among the total twenty high AFP patients, while 32 (23.2%) patients displayed liver metastasis among the total 138 low AFP patients, which led to a statistical difference (Table 1, *P* = 0.011). The ORRs of the low and high AFP groups were comparable (15.9% vs. 20.0%, *P* = 0.995), while the DCR showed statistical significance with 87.7% for the low AFP group and 50.0% for the high AFP group (*P* < 0.001, Table 2). These findings suggest that baseline AFP levels could affect the therapeutic efficacy of anti-PD-1 antibodies in advanced gastric cancer patients.

### AFP associated with PFS and OS of AGC patients

**Fig. 1:**
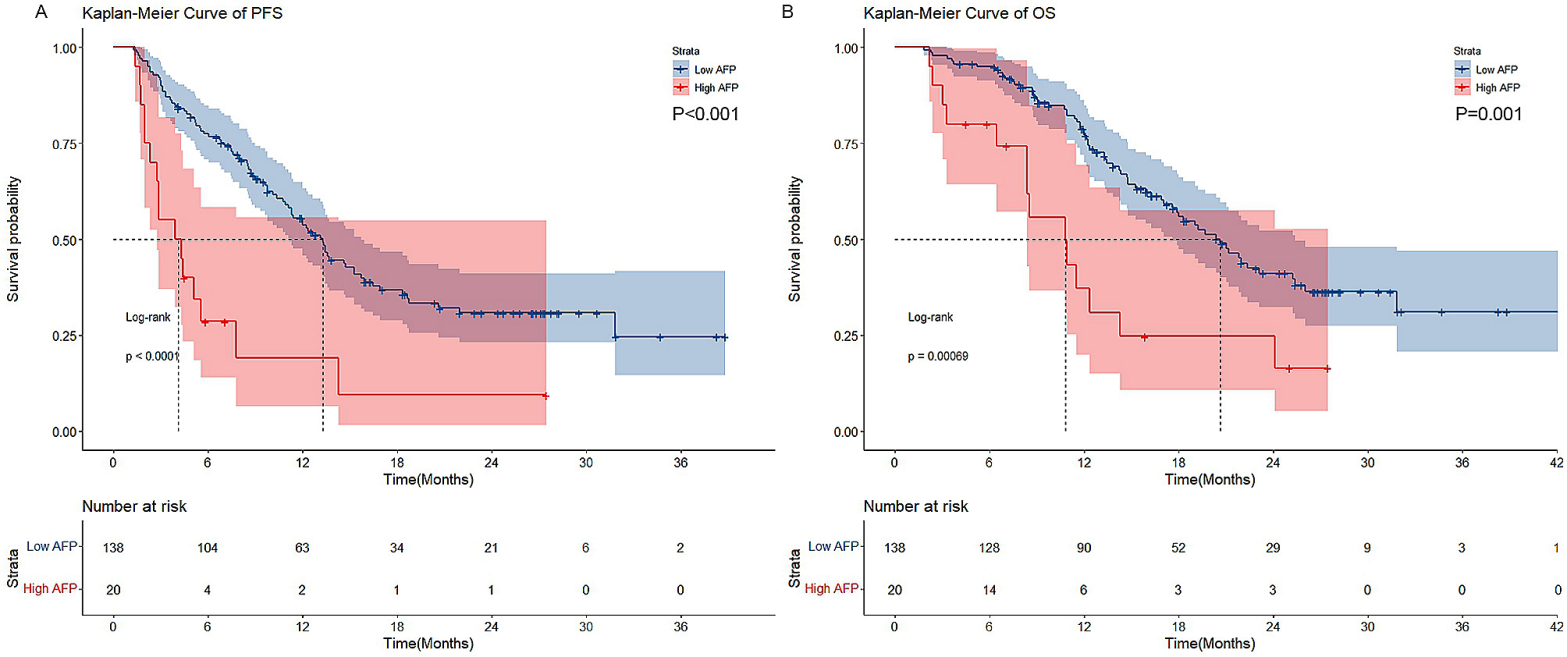
The association of AFP on the prognosis of advanced gastric cancer patients. **(A)** The Kaplan–Meier curve of progression-free survival (PFS). **(B)** The Kaplan–Meier curve of overall survival (OS)

Figure 1A displays the Kaplan-Meier curves comparing the PFS between a low AFP group and a high AFP group. Significantly, the low AFP group exhibited a substantial increase in median PFS compared to the high AFP group (13.300 months vs. 3.933 months *P* < 0.001). During univariate analysis of the PFS, the baseline AFP levels (*P* < 0.001, HR: 3.419, 95% CI: 1.973–5.925, Table 3), TNM stage (*P* = 0.001, HR: 2.092, 95% CI: 1.335–3.277), and treatment lines (*P* < 0.001, HR: 2.751, 95% CI: 1.665–4.546) were associated with PFS in AGC patients. Conversely, factors such as age, gender, ECOG PS, status of HER2, status of EBV, surgical history, liver metastases, and treatment regimen did not exert a significant impact on PFS (*P* > 0.05). The subsequent multivariate analysis confirmed that a higher baseline AFP level independently correlated with a shorter PFS (*P* < 0.001, HR = 2.891, 95% CI = 1.648–5.070, Table 4). Additionally, the TNM stage (*P* = 0.006, HR: 1.888, 95% CI: 1.197–2.978) and number of treatment lines (*P* < 0.001, HR: 2.743, 95% CI: 1.657–4.541) also independently influenced PFS (Table 4).

**Table 3:** Univariate analyses of PFS and OS

Covariate		PFS			OS	
	HR	95%CI	***P***	HR	95%CI	***P***
AFP						
Low	Reference			Reference		
High	3.419	1.973–5.925	0.000	2.631	1.472–4.703	0.001
Age						
< 60	Reference			Reference		
≥ 60	0.784	0.523–1.175	0.239	0.911	0.580–1.431	0.686
Gender						
Male	Reference			Reference		
Female	1.116	0.710–1.756	0.634	0.893	0.543–1.468	0.655
ECOG PS						
0–1	Reference			Reference		
2–3	1.421	0.958–2.107	0.081	1.264	0.817–1.958	0.293
CPS						
< 5	Reference			Reference		
≥ 5	0.677	0.427–1.073	0.097	0.687	0.413–1.142	0.148
HER2 status						
Negative	Reference			Reference		
Positive	0.977	0.564–1.693	0.934	1.121	0.618–2.033	0.708
EBV status						
Negative	Reference			Reference		
Positive	0.776	0.391–1.542	0.469	0.767	0.353–1.669	0.504
Surgical history						
No	Reference			Reference		
Yes	0.832	0.540–1.280	0.402	0.759	0.467–1.235	0.267
TNM stage						
III	Reference			Reference		
IV	2.092	1.335–3.277	0.001	2.291	1.369–3.835	0.002
Treatment regimen						
ICI plus chemotherapy	Reference		0.215	Reference		0.529
ICI plus targeted therapy	1.647	0.937–2.893	0.083	1.222	0.646–2.314	0.537
ICI plus chemotherapy and targeted therapy	1.175	0.716–1.930	0.523	1.332	0.788–2.250	0.285
Treatment lines						
1–2	Reference			Reference		
≥ 3	2.751	1.665–4.546	0.000	1.992	1.150–3.449	0.014
Liver metastases						
No	Reference			Reference		
Yes	1.279	0.821–1.990	0.276	1.316	0.811–2.135	0.266

PFS: progression-free survival; OS: overall survival; AFP: alpha-fetoprotein; ECOG PS: Eastern Cooperative Oncology Group Performance Status; CPS: Combined Positive Score; HER2: human epidermal growth factor receptor 2; EBV: Epstein–Barr virus

**Table 4:** Multivariate analyses of PFS and OS with the Cox proportional hazards model

	Variables	Total No.		PFS			OS	
			HR	95%CI	***P***	HR	95%CI	***P***
Group								
	Low AFP	138	Reference			Reference		
	High AFP	20	2.891	1.648–5.070	0.000	2.198	1.207–4.003	0.010
TNM stage								
	III	55	Reference			Reference		
	IV	103	1.888	1.197–2.978	0.006	2.124	1.264–3.567	0.004
Treatment lines								
	1–2	138	Reference			Reference		
	≥ 3	20	2.743	1.657–4.541	0.000	1.575	0.892–2.781	0.118

PFS: progression-free survival; OS: overall survival; AFP: alpha-fetoprotein

The low AFP group was associated with longer overall survival than the high baseline AFP group (median OS 20.633 months vs. 10.800 months, *P* = 0.001, Fig. 1B). The univariate analysis found that baseline AFP levels (*P* = 0.001, HR: 2.631, 95% CI: 1.472–4.703), TNM stage (*P* = 0.002, HR: 2.291, 95% CI: 1.369–3.835), and treatment lines (*P* = 0.014, HR: 1.992, 95% CI: 1.150–3.449) were significantly associated with the OS of patients with AGC (Table 3). In addition, multivariate analysis showed that baseline AFP levels (*P* = 0.010, HR: 2.198, 95% CI: 1.207–4.003) and TNM stage (*P* = 0.004, HR: 2.124, 95% CI: 1.264–3.567) were independent prognostic factors for OS of AGC patients, which indicated that the risk of death in the high AFP group was 2.198-fold higher than the low AFP group (Table 4). These data demonstrated that baseline AFP levels were associated with the PFS and the OS of AGC patients.

The actual concentration of AFP in the AFP high group of patients was shown in Table 5. The relationship between the actual AFP levels in the AFP high group and PFS/OS was evaluated using the Spearman correlation test. A negative correlation trend without statistical difference was found (PFS: *r*=-0.240, *p* = 0.308; OS: *r*=-0.239, *p* = 0.310).

**Table 5:** Actual AFP concentration in patients with high AFP levels

No.	AFP concentration(ng/ml)	PFS (months)	OS (months)
1	≥ 1210.00	2.77	12.37
2	21.58	1.97	3.27
3	140.40	2.33	10.90
4	406.30	1.70	2.40
5	84.05	2.90	11.53
6	≥ 1210.00	1.37	2.20
7	96.70	14.30	14.30
8	1207.00	5.57	8.53
9	31.16	1.73	15.90
10	26.03	2.87	8.40
11	30.00	7.80	24.10
12	21.00	27.47	27.47
13	234.00	3.93	10.80
14	≥ 1210.00	1.97	3.03
15	528.00	5.10	6.47
16	1192.00	7.10	7.10
17	≥ 1210.00	4.43	25.03
18	29.05	4.33	8.40
19	≥ 1210.00	4.50	4.50
20	28.45	5.87	5.87

AFP: alpha-fetoprotein

### Association between MSI status and ORR in AGC patients

A total of 122 patients with confirmed evaluable MSI status including 116 (95.1%) MSS/MSI-low and 6 (4.9%) MSI-high were evaluated for their association with treatment efficiency of ICI. The distribution frequency for MSI status was not different between the high AFP and low AFP groups (data not shown). The DCR was 79.3% for MSS/MSI-low whereas 100% for MSI-high. The ORR was 13.8% for MSS/MSI-low and 50% for MSI-high with statistical difference (*P* = 0.048, Table 6). These findings suggest that the MSI-high group has a higher ORR in advanced gastric cancer patients. In the subsequent subgroup analysis on 116 patients with MSS/MSI-low, the multivariate analysis confirmed that higher baseline AFP levels were independently associated with shorter PFS (*P* = 0.001, HR = 2.930, 95% CI = 1.540–5.572) and shorter OS (*P* = 0.007, HR = 2.550, 95% CI = 1.299–5.007).

**Table 6:** MSI status associated with immunotherapy response in gastric cancer patients

Response	Total No.	MSS/MSI-Low	MSI-High	***P***
PD	24	24	0	
SD	79	76	3	
PR	18	15	3	
CR	1	1	0	
ORR	15.6% (95% CI: 9.0-22.1%)	13.8% (95%CI: 7.4-20.2%)	50% (95% CI: -7.5-107.5%)	0.048
DCR	80.3% (95%CI: 73.2-87.5%)	79.3% (95%CI: 71.8-86.8%)	100% (95%CI: 100.0%-100.0%)	0.214

CR: complete response; PR: partial response; SD: stable disease; PD: progressive disease; DCR: disease control rate; ORR: objective response rate; MSI: microsatellite instability

### Subgroup analysis for patients receiving ICI plus chemotherapy

The subgroup analysis for ICI plus chemotherapy patients was performed, the distribution frequency for clinical features including age, gender, ECOG PS, CPS, HER2 status, EBV status, TNM stage, surgical history, treatment regimen, treatment lines, and disease status were not different between the low AFP group and the high AFP group except for liver metastases (*P* = 0.023) (Supplementary Table S1). During the univariate analysis of PFS and OS, baseline AFP levels (*P* = 0.002, HR: 2.942, 95% CI: 1.487–5.822 for PFS, *P* = 0.006, HR: 2.796, 95% CI: 1.352–5.782 for OS, Supplementary Table S2) and TNM stage (*P* = 0.004, HR: 2.184, 95% CI: 1.283–3.717 for PFS, *P* = 0.003, HR: 2.589, 95% CI: 1.387–4.834 for OS, Supplementary Table S2) were found to be associated with both PFS and OS. Subsequent multivariate analysis confirmed that higher baseline AFP levels independently correlated with shorter PFS (*P* = 0.021, HR = 2.279, 95% CI = 1.133–4.585, Supplementary Table S3) and shorter OS (*P* = 0.013, HR = 2.530, 95% CI = 1.217–5.258, Supplementary Table S3).

### Results of the propensity score-matched analysis

To decrease the effect of potential confounding factors, we conducted a one-to-four propensity score matching analysis. Propensity score matching was performed based on three variables that were identified as the most important for the final matching: TNM stage, ECOG PS, and liver metastases. This resulted in the inclusion of 18 patients (42.9%) in the high AFP group and 24 patients (57.1%) in the low AFP group. The clinical characteristics of the individuals after propensity score matching are presented in Table S4. Following propensity score matching, a log rank test revealed that the high AFP group exhibited a decrease in median PFS(12.000 months vs. 3.930 months, *P* = 0.011) and median OS (19.270 months vs. 10.800 months, *P* = 0.036) compared to the low AFP group.

## Discussion

In recent years, there has been a shift in the treatment approach for AGC, moving from chemotherapy to molecular targeted therapy and, more recently, to immunotherapy. The utilization of immunotherapy has transitioned from being a third-line therapy to becoming a preferred first-line treatment option for AGC. Due to the lack of predictive biomarkers for ICI therapy in AGC, we conducted a retrospective analysis to assess the prognostic significance of the baseline serum AFP level, which showed the prediction value for AGC immunotherapy, in AGC patients undergoing ICI treatment. Our study revealed a significant association for baseline AFP levels with DCR, PFS and OS in AGC individuals. As far as we are aware, this study is the first report for the association of AFP and ICI efficency in AGC patients.

The precise mechanism by which AFP levels influence the efficacy of ICIs in tumor patients remains unclear. Previous studies have reported AFP could directly promote the proliferation and growth of cancer cells, as well as block cell apoptosis [27]. In hepatocytes, AFP binds to its membrane receptor to activate the cAMP-PKA cellular pathway as well as enhance the expression of RAS, c-jun, and c-fos oncogenes, thereby facilitates the S phase transition of cell cycle and stimulates angiogenesis (proliferation) [28, 29]. AFP could positively regulate cell proliferation and enhance the apoptosis resistance via effect on transforming growth factor-β(TGF-β) andp53/Bax/caspase-3 signaling pathway in HepG2 cells [30]. In addition, AFP could both activate the PI3K/P-AKT/mTOR cellular pathway and stimulate the cancer cell growth by binding with phosphatase and tensin homolog (PTEN) [27, 28, 31].

As for the immunotherapy, AFP inhibits not only the differentiation of monocytes into fully functional dendritic cells but the dendritic cells in presenting foreign antigens to CD8 + lymphocytes through the MHC pathways [32, 33]. In addition, AFP reduces the production of Toll-like receptor 4 (TLR4) on the surface of DCs so as to block the production of pro-inflammatory cytokines including interleukin 12(IL-12) and tumor necrosis factor-α(TNF-α), which can stimulate the overgeneration of CD4 + and cytotoxic CD8 + lymphocytes in immunotherapy [21]. Furthermore, AFP induces the differentiation of ThCD4 + lymphocytes into Tregs to negatively regulate the immunotherapy through the altered tolerogenic DCs [28, 34]. Moreover, AFP causes apoptosis of NK cells or inhibits their activation by dendritic cells [35]. AFP might weaken the efficacy of ICI by the above mechanisms in AGC patients.

Our data implied that AFP is an important target for ICI treatment, AFP inhibition cooperating with ICI might improve the treatment efficacy of AGC patients with elevated AFP. The fact that the AFP-targeted CAR T-cell therapy is ongoing for hepatocellular carcinoma treatment provides the possibility for treating AFP elevated AGC patients with AFP targeting [36, 37].

Our study has certain limitations that should be acknowledged. Firstly, it is a retrospective analysis with smaple size that was conducted at a single center. Multi-center, high-quality, large sample size prospective research need to be further implemented. Secondly, due to the limited pathological tissue, we did not conduct immunohistochemical staining of AFP levels in cancer tissue, although its expression in tissues is positively correlated with its secretion into the blood [38, 39]. Thirdly, we didn’t measure the lymphocyte density in tumor tissues to explore their correlation with AFP levels, the basic experiments with humanized animal model should be performed to evaluate the changes of tumor microenvironment (especially immune cells) upon AFP induction. Lastly, it is worth mentioning that almost all of our patients received combination therapy. Thus, the potential impact of combined medication on patient prognosis was not entirely ruled out in this study. However, our study results have confirmed the significance of AFP as a biomarker in assessing the effectiveness of ICIs treatment, thereby encouraging other scientists to investigate predictors of efficacy in other tumors treated with ICIs.

## Conclusions

Baseline AFP levels may predict immune checkpoint inhibitor treatment efficacy in AGC patients.

### Electronic supplementary material

Below is the link to the electronic supplementary material.

Supplementary Material 1

## Data Availability

The original contributions presented in the study are included in the article. Further inquiries can be directed to the corresponding author.

## Abbreviations

ICIs: Immune checkpoint inhibitors
NK: Natural killer
AFP: Alpha-fetoprotein
AGC: Advanced gastric cancer
ORR: Objective response rate
DCR: Disease control rate
PFS: Progression-free survival
OS: Overall survival
AGC: Advanced gastric cancer
Hp: Helicobacter pylori
PD-1: Programmed cell death 1
PD-L1: Programmed cell death ligand 1
CTLA-4: Cytotoxic T-lymphocyte antigen 4
MSI: Microsatellite instability
MMR: Mismatch repair
ECOG PS: Eastern Cooperative Oncology Group Performance Status
CPS: Combined positive score
HER2: Status of human epidermal growth factor receptor 2
EBV: Status of Epstein-Barr virus
CR: Complete response
PR: Partial response
SD: Stable disease
TGF-β: Transforming growth factor-β
PTEN: Phosphatase and tensin homolog
TLR4: Toll-like receptor 4
IL-12: Interleukin 12
TNF-α: Tumor necrosis factor-α
