# Supplementary material for: Alpha-fetoprotein predicts the treatment efficacy of immune checkpoint inhibitors for gastric cancer patients
Source: BMC Cancer. 2024 Feb 26;24:266. doi: 10.1186/s12885-024-11999-z (PMC10895833; doi:10.1186/s12885-024-11999-z)
Supplement: Supplementary file 1 — Supplementary Material 1 [file 12885_2024_11999_MOESM1_ESM.docx]

TABLE S1 Characteristics of advanced gastric cancer patients with different AFP levels in the ICI plus chemotherapy group.

| Covariate | Total No. (%) | Low AFP (%) | High AFP (%) | P |
| --- | --- | --- | --- | --- |
| Total | 110 | 97 | 13 |  |
| Age |  |  |  |  |
| <60 | 34(30.9) | 29（29.9） | 5(38.5) | 0.758 |
| ≥60 | 76(69.1) | 68（70.1） | 8(61.5) |  |
| Gender |  |  |  |  |
| Male | 85(77.3) | 74（76.3） | 11(84.6) | 0.749 |
| Female | 25(22.7) | 23（23.7） | 2(15.4) |  |
| ECOG PS |  |  |  |  |
| 0-1 | 68(61.8) | 62(63.9) | 6(46.2) | 0.350 |
| 2-3 | 42(38.2) | 35(36.1) | 7(53.8) |  |
| CPS |  |  |  |  |
| <5 | 82(74.5) | 71(73.2) | 11(84.6) | 0.583 |
| ≥5 | 28(25.4) | 26(26.8) | 2(15.4) |  |
| HER2 status |  |  |  |  |
| Negative | 103(93.6) | 92(94.8) | 11(84.6) | 0.193 |
| Positive | 7(6.4) | 5(5.2) | 2(15.4) |  |
| EBV status |  |  |  |  |
| Negative | 100(90.9) | 87(89.7) | 13(100.0) | 0.484 |
| Positive | 10(9.1) | 10(10.3) | 0(0.00) |  |
| Surgical history |  |  |  |  |
| No | 79(71.8) | 68(70.1) | 11(84.6) | 0.445 |
| Yes | 31(28.2) | 29(29.9) | 2(15.4) |  |
| TNM stage |  |  |  |  |
| III | 46(41.8) | 43(44.3) | 3(23.1) | 0.145 |
| IV | 64(58.2) | 54(55.7) | 10(76.9) |  |
| Treatment lines |  |  |  |  |
| 1-2 | 106(96.4) | 93(84.5) | 13(100.0) | 1.000 |
| ≥3 | 4(3.6) | 4(15.5) | 0(0.00) |  |
| Liver metastases |  |  |  |  |
| No | 89(80.9) | 82(84.5) | 7(53.8) | 0.023 |
| Yes | 21(19.1) | 15(15.5) | 6(46.2) |  |

AFP, alpha-fetoprotein; ECOG PS, Eastern Cooperative Oncology Group Performance Status; CPS, Combined Positive Score; HER2, human epidermal growth factor receptor 2; EBV, Epstein–Barr virus.

TABLE S2 Univariate analyses of PFS and OS in the subgroup of ICI plus chemotherapy gastric cancer patients.

| Covariate |  | PFS |  |  | OS |  |
| --- | --- | --- | --- | --- | --- | --- |
|  | HR | 95%CI | P | HR | 95%CI | P |
| AFP |  |  |  |  |  |  |
| Low | Reference |  |  | Reference |  |  |
| High | 2.942 | 1.487- 5.822 | 0.002 | 2.796 | 1.352-5.782 | 0.006 |
| Age |  |  |  |  |  |  |
| <60 | Reference |  |  | Reference |  |  |
| ≥60 | 0.674 | 0.403- 1.125 | 0.131 | 0.714 | 0.397-1.282 | 0.259 |
| Gender |  |  |  |  |  |  |
| Male | Reference |  |  | Reference |  |  |
| Female | 0.983 | 0.543-1.779 | 0.955 | 0.903 | 0.461- 1.768 | 0.766 |
| ECOG PS |  |  |  |  |  |  |
| 0-1 | Reference |  |  | Reference |  |  |
| 2-3 | 1.455 | 0.886-2.387 | 0.138 | 1.119 | 0.632-1.980 | 0.701 |
| CPS |  |  |  |  |  |  |
| <5 | Reference |  |  | Reference |  |  |
| ≥5 | 0.626 | 0.345-1.135 | 0.122 | 0.614 | 0.312-1.210 | 0.159 |
| HER2 status |  |  |  |  |  |  |
| Negative | Reference |  |  | Reference |  |  |
| Positive | 0.848 | 0.266-2.706 | 0.781 | 0.771 | 0.187-3.175 | 0.718 |
| EBV status |  |  |  |  |  |  |
| Negative | Reference |  |  | Reference |  |  |
| Positive | 0.770 | 0.331-1.790 | 0.543 | 0.833 | 0.329-2.106 | 0.700 |
| Surgical history |  |  |  |  |  |  |
| No | Reference |  |  | Reference |  |  |
| Yes | 0.899 | 0.521-1.553 | 0.703 | 0.712 | 0.371-1.368 | 0.308 |
| TNM stage |  |  |  |  |  |  |
| III | Reference |  |  | Reference |  |  |
| IV | 2.184 | 1.283-3.717 | 0.004 | 2.589 | 1.387-4.834 | 0.003 |
| Treatment lines |  |  |  |  |  |  |
| 1-2 | Reference |  |  | Reference |  |  |
| ≥3 | 2.438 | 0.880-6.753 | 0.087 | 2.680 | 0.815-8.810 | 0.105 |
| Liver metastases |  |  |  |  |  |  |
| No | Reference |  |  | Reference |  |  |
| Yes | 1.461 | 0.793-2.693 | 0.224 | 1.846 | 0.940-3.622 | 0.075 |

PFS, progression-free survival; OS, overall survival; AFP, alpha-fetoprotein; ECOG PS, Eastern Cooperative Oncology Group Performance Status; CPS, Combined Positive Score; HER2, human epidermal growth factor receptor 2; EBV, Epstein–Barr virus.

TABLE S3 Multivariate analyses of PFS and OS in the subgroup of ICI plus chemotherapy gastric cancer patients.

|  | Variables | Total No. |  | PFS |  |  | OS |  |
| --- | --- | --- | --- | --- | --- | --- | --- | --- |
|  |  |  | HR | 95%CI | P | HR | 95%CI | P |
| Group |  |  |  |  |  |  |  |  |
|  | Low AFP | 97 | Reference |  |  |  |  |  |
|  | High AFP | 13 | 2.279 | 1.133-4.585 | 0.021 | 2.530 | 1.217-5.258 | 0.013 |
| TNM stage |  |  |  |  |  |  |  |  |
|  | III | 46 | Reference |  |  |  |  |  |
|  | IV | 64 | 2.087 | 1.199-3.633 | 0.009 | 2.725 | 1.436-5.172 | 0.002 |

PFS, progression-free survival; OS, overall survival; AFP, alpha-fetoprotein.

TABLE S4. Characteristics of the study population after propensity score matching.

| Covariate | Total No. (%) | Low AFP (%) | High AFP (%) | P |
| --- | --- | --- | --- | --- |
| Total | 42 | 24 | 18 |  |
| Gender |  |  |  |  |
| Male | 34(81.0) | 18(75.0) | 16(88.9) | 0.461 |
| Female | 8(19.0) | 6(25.0) | 2(11.1) |  |
| ECOG PS |  |  |  |  |
| 0-1 | 20(47.6) | 12(50.0) | 8(44.4) | 0.721 |
| 2-3 | 22(52.4) | 12(50.0) | 10(55.6) |  |
| TNM stage |  |  |  |  |
| III | 12(28.6) | 8(33.3) | 4(22.2) | 0.430 |
| IV | 30(71.4) | 16(66.7) | 14(77.8) |  |
| Liver metastases |  |  |  |  |
| No | 26(61.9) | 16(66.7) | 10(55.6) | 0.463 |
| Yes | 16(38.1) | 8(33.3) | 8(44.4) |  |

AFP, alpha-fetoprotein; ECOG PS, Eastern Cooperative Oncology Group Performance Status.
